# Rubber hand illusion induced by touching the face ipsilaterally to a deprived hand: evidence for plastic “somatotopic” remapping in tetraplegics

**DOI:** 10.3389/fnhum.2014.00404

**Published:** 2014-06-10

**Authors:** Michele Scandola, Emmanuele Tidoni, Renato Avesani, Giovanni Brunelli, Salvatore M. Aglioti, Valentina Moro

**Affiliations:** ^1^IRCCS Fondazione S. LuciaRome, Italy; ^2^SCNLab, Department of Psychology, University “La Sapienza” of RomeRome, Italy; ^3^NPsy-Lab.VR, Department of Philosophy, Education and Psychology, University of VeronaVerona, Italy; ^4^Department of Rehabilitation, Sacro Cuore HospitalNegrar, Verona, Italy

**Keywords:** spinal cord injury, rubber hand illusion, somatosensory plasticity, body representation, tetraplegia, face-hand remapping

## Abstract

**Background:** Studies in animals and humans indicate that the interruption of body-brain connections following spinal cord injury (SCI) leads to plastic cerebral reorganization.

**Objective:** To explore whether inducing the Rubber Hand Illusion (RHI) via synchronous multisensory visuo-tactile bodily stimulation may reveal any perceptual correlates of plastic remapping in SCI.

**Methods:** In 16 paraplegic, 16 tetraplegic and 16 healthy participants we explored whether RHI may be induced by tactile stimuli involving not only the left hand but also the left hemi-face. Touching the participants actual hand or face was either synchronous or asynchronous with tactile stimuli seen on a rubber hand. We assessed two components of the illusion, namely perceived changes in the real hand in space (indexed by proprioceptive drift) and ownership of the rubber hand (indexed by subjective responses to an ad-hoc questionnaire).

**Results:** Proprioceptive drift and ownership were found in the healthy group only in the condition where the left real and fake hand were touched simultaneously. In contrast, no drift was found in the SCI patients who, however, showed ownership after both synchronous and asynchronous hand stroking. Importantly, only tetraplegics showed the effect also after synchronous face stroking.

**Conclusions:** RHI may reveal plastic phenomena in SCI. In hand representation-deprived tetraplegics, stimuli on the face (represented contiguously in the somatic and motor systems), drive the sense of hand ownership. This hand-face remapping phenomenon may be useful for restoring a sense of self in massively deprived individuals.

## 1. Introduction

Spinal cord injuries (SCI) cause an irreversible disconnection between the body and the brain. This disconnection implies a deprivation of somatosensory input to and motor output from the brain. The extent of this deprivation depends on the level and completeness of the lesion. While cervical SCI leads to tetraplegia, a clinical condition with impaired sensory-motor functions in both upper and lower limbs, SCI below the seventh cervical spinal cord segment leads to paraplegia, where deficits affect lower but not upper limbs.

Studies indicate that sensorimotor deprivation in SCI may induce alterations in the bodily-self as indexed by the Rubber Hand Illusion (RHI), where the induction of a visuo-tactile conflict allows rapid changes in body-ownership (Botvinick and Cohen, 1998). In initial studies of the RHI healthy individuals were asked to look at a rubber hand that was stroked by the examiner, synchronously or asynchronously with their hidden from view real hand. It appeared that only during synchronous stimulation was the rubber hand perceived as part of the participants' own body (index of ownership of an artificial hand) and the position of the real hand was perceived as having shifted toward the rubber hand (“proprioceptive drift,” index of illusory perception of body in space) (Botvinick and Cohen, 1998; Ehrsson et al., 2005; Tsakiris and Haggard, 2005; Longo et al., 2008; Mohan et al., 2012; Schaefer et al., 2013).

The first time that the RHI paradigm was applied to SCI participants demonstrated that SCI did not alter subjective indices of the illusory hand ownership (Lenggenhager et al., 2012). However, proprioceptive drift was found to be deviated only in subjects with defective hand perception, suggesting that plasticity-related cortical changes might influence the dynamics of the bodily-self (Lenggenhager et al., 2012). The RHI has subsequently been used to induce a restoration of impaired hand somatosensivity in two SCI patients (Lenggenhager et al., 2013).

In many previous papers the “proprioceptive drift” is defined as an objective measure of the RHI. We contend it has to be considered as a subjective response. We suggest that this measure is at least as subjective as the point of subjective equivalence (PSE) (Gescheider, 1997), i.e., the index widely used in psychophysics research which is computed starting from the participants' answers about the perception or the lack of perception of a stimulus, or the perceived difference between two sensory stimuli. For these reasons in this study we defined, the “proprioceptive drift” as a subjective index of perception of the body in space. We propose that psychogalvanic response (Armel and Ramachandran, 2003; Ferri et al., 2013), the change in temperature (Moseley et al., 2008), etc. are considered objective indexes in rubber-hand or full-body illusion studies.

Brain reorganization induced by a reduction in somatosensory and motor inputs has been demonstrated in studies on animals and humans (Nahum et al., 2013). Crucial for the present research, this type of reorganization may follow topographic rules. For example, a single cell recording study on monkeys deprived of somatosensory input due to an extended dorsal rhizotomy, demonstrated that the cortical territories formerly mapping the de-afferented skin regions (e.g., the hand) were driven by inputs coming from brain regions with adjacent, intact representation (e.g., the face) (Pons et al., 1991). Evidence for the perceptual correlates of this topographic remapping process has been provided by studies on individuals with upper-limb (Ramachandran et al., 1992; Aglioti et al., 1997), lower-limb (Aglioti et al., 1994a) or breast (Aglioti et al., 1994b) amputation and phantom perception of the lost body part. In particular, tactile stimuli on the face ipsilaterally to hand or finger amputations induced in a considerable number of patients the sensation of being touched not only on the face but also on the phantom hand (Ramachandran et al., 1992) or finger (Aglioti et al., 1997). Consistent and precise, but topographically disorganized, double sensations were evoked by tactile stimuli applied to the contralesional hypoaesthesic hand in a patient with a selective lesion involving hand representation in the primary somatosensory cortex (Aglioti et al., 1999).

Based on the notion that the somatosensory and motor brain representation of the face and the hand are contiguous, double sensations were interpreted as an index of remapping of the face on the de-afferented hand representation. The inherent link between hand and face representations is also supported by studies on healthy subjects in whom complete temporary anaesthesia of the thumb rapidly induced the sensation that the size of the lips increased by up to 50% (Gandevia and Phegan, 1999). Neurophysiological evidence for face-hand remapping has been provided by an EEG study documenting that tactile stimulation of the hand activates the cortical representation of the face in people who had undergone cosmetic injections of botulinum toxin to treat wrinkles (Haenzi et al., 2014). As previously mentioned, deprivation related neuroplasticity may also be at play after spinal cord lesions. What remains unknown is whether the perceptual correlates of the plasticity found in SCI follows erratic rules (Moore et al., 2000) or may also occur according to somewhat topographic organization.

We explored this issue by applying a novel version of the classic RHI paradigm to healthy, paraplegic, and tetraplegic people. Based on the study on monkeys with cervical SCI who showed an expansion of the face representation in the primary and non-primary somatosensory cortex toward nearby areas (Tandon et al., 2009), we hypothesized that tetraplegics, but not paraplegics and healthy people, would experience the RHI after stimulation of their cheek synchronously with rubber hand stimulation. We modified two main aspects of the classic RHI paradigm (Botvinick and Cohen, 1998). The first concerns the stimulation which was applied not only to the participants real hand but also to their cheek. The second is that rubber hand and real hand were vertically aligned with the former in a higher position with respect to the latter. We measured any possible vertical drift in the perceived position of the participants real hand (Bekrater-Bodmann et al., 2012). We expected only the tetraplegics to show indices of RHI in the Face- Synchronous condition, due to possible mechanisms of plasticity in the somatosensory networks. Instead, in the paraplegics and healthy individuals, we expected to replicate RHI effects exclusively following Synchronous Hand-stimulation.

## 2. Materials and methods

### 2.1. Participants

Sixteen tetraplegics, sixteen paraplegics and sixteen neurologically healthy participants participated in the study. All participants were right-handed and had normal or corrected-to-normality vision. The three groups were gender-, age- and education-matched (log-linear analysis on gender data: χ^2^_(2)_ = 2.1646, *p* = 0.34, One-Way ANOVA on age data: *F*_(2,45)_ = 0.90, *p* = 0.41, One-Way ANOVA on education, converted in a numerical value from 1 = junior school to 4 = bachelor degree: *F*_(2,45)_ = 0.84, *p* = 0.44).

For all SCI participants, the Neurological Level of Injury (NLI) and the American Spinal Injury Association Impairment Scale (AIS, index of completeness of lesion) were collected, according to the International Standards for Neurological Classification of Spinal Cord Injury (Kirshblum et al., 2011).

The NLI is defined as the more rostral spinal cord segment where both the sensory and motor functions are spared. This does not exclude the possibility that some motor or sensory functions are spared below this level. This indeed is what may happen in cases of incomplete lesions (Kirshblum et al., 2011). The AIS gives information about the completeness of lesions. The AIS score (ranging from A to E in a decreasing order of impairment) is calculated on the basis of motor and sensory functions preserved at the level of sacral segments S4-S5 (that are the most caudals) (Kirshblum et al., 2011). Only SCI participants with scores of “A” (absence of sensory and motor functions at S4-S5) or “B” (spared sensory but not motor functions at S4-S5) in the AIS were recruited in this study and assigned to the Tetraplegics and Paraplegics groups according to their NLI (upper or lower C7 level, respectively). In the Tetraplegics group, left-hand tactile perception was tested. Taking into consideration both the definitions of NLI and AIS, it is clear that, also even if the NLI is very high and the AIS is “A,” there is the possibility of spared sensory sensations in the left hand and, on the other side, that a low NLI and an AIS of “B” does not guarantee spared tactile sensations on the left hand. Furthermore, the Spinal Cord Independence Measure-III (SCIM-3) was administered in order to quantify the degree of functional autonomy (Invernizzi et al., 2010). Demographical and clinical data are reported in Table 1. The study was approved by the Ethics committee of the Province of Verona (Prot. N. 40378) and was conducted in accordance with the ethical standards of the 1964 Declaration of Helsinki. All participants gave their informed consent.

**Table 1:** **Demographic and clinical information relative to the SCI subjects**.

**ID**	**Gender**	**Age (Years)**	**Interval (Years)**	**SCIM-3**	**NLI**	**AIS**	**Hand tactile sensation**
T1	M	71	1	10	C4	A	−
T2	F	50	12	31	C5	B	+
T3	M	25	3	39	C6	A	+
T4	M	50	31	36	C5	A	−
T5	M	42	14	25	C4	B	−
T6	M	55	31	36	C5	B	−
T7	M	40	1	25	C5	A	−
T8	M	65	1	47	C6	A	+
T9	F	42	9	28	C6	A	+
T10	F	39	21	16	C6	B	+
T11	F	65	15	18	C4	A	+
T12	M	50	26	20	C6	A	+
T13	F	29	14	20	C4	B	−
T14	F	41	26	54	C6	B	+
T15	M	52	2	63	C5	B	+
T16	M	18	5	66	C6	B	+
*Mean*	*F*: 6	45.88	13.25	33.38			
*SD*	*M*: 10	14.53	10.92	16.76			
*Range*		18–71	1–31	10–66	C4–C6		
P1	M	33	1	77	T4	A	+
P2	F	44	10	75	T1	A	+
P3	M	62	40	75	L1	A	+
P4	M	31	14	72	L4	A	+
P5	M	54	30	75	L3	A	+
P6	M	60	4	67	T8	A	+
P7	M	79	28	26	T12	A	+
P8	M	47	27	73	T11	B	+
P9	F	53	29	74	T10	A	+
P10	M	47	3	75	T7	A	+
P11	M	45	27	77	T12	B	+
P12	M	48	12	61	T5	A	+
P13	F	26	10	75	T6	A	+
P14	M	60	5	65	T4	A	+
P15	F	51	32	75	T12	A	+
P16	M	60	24	71	T6	A	+
*Mean*	*F*: 4	50.00	18.50	69.56			
*SD*	*M*: 12	13.22	12.38	12.45			
*Range*		26–79	1–40	26–77	T1–L4		
*Healthy mean*	*F*: 8	43.07					
*Healthy SD*	*M*: 8	16.88					
*Healthy range*		22–80					

*Data referring to Tetraplegics and Paraplegics participants are reported in the upper and central parts of the table, respectively. In the lower part, the mean and SD data referring to the healthy group are indicated. NLI, Neurological Level of Injury; Interval, time interval between lesion onset and experimental session; AIS, ASIA Impairment Scale; SCIM-3, Spinal Cord Independence Measure-III Edition; “+,” absence of deficit; “−,” presence of deficit*.

### 2.2. Materials and procedure

**Figure 1:**
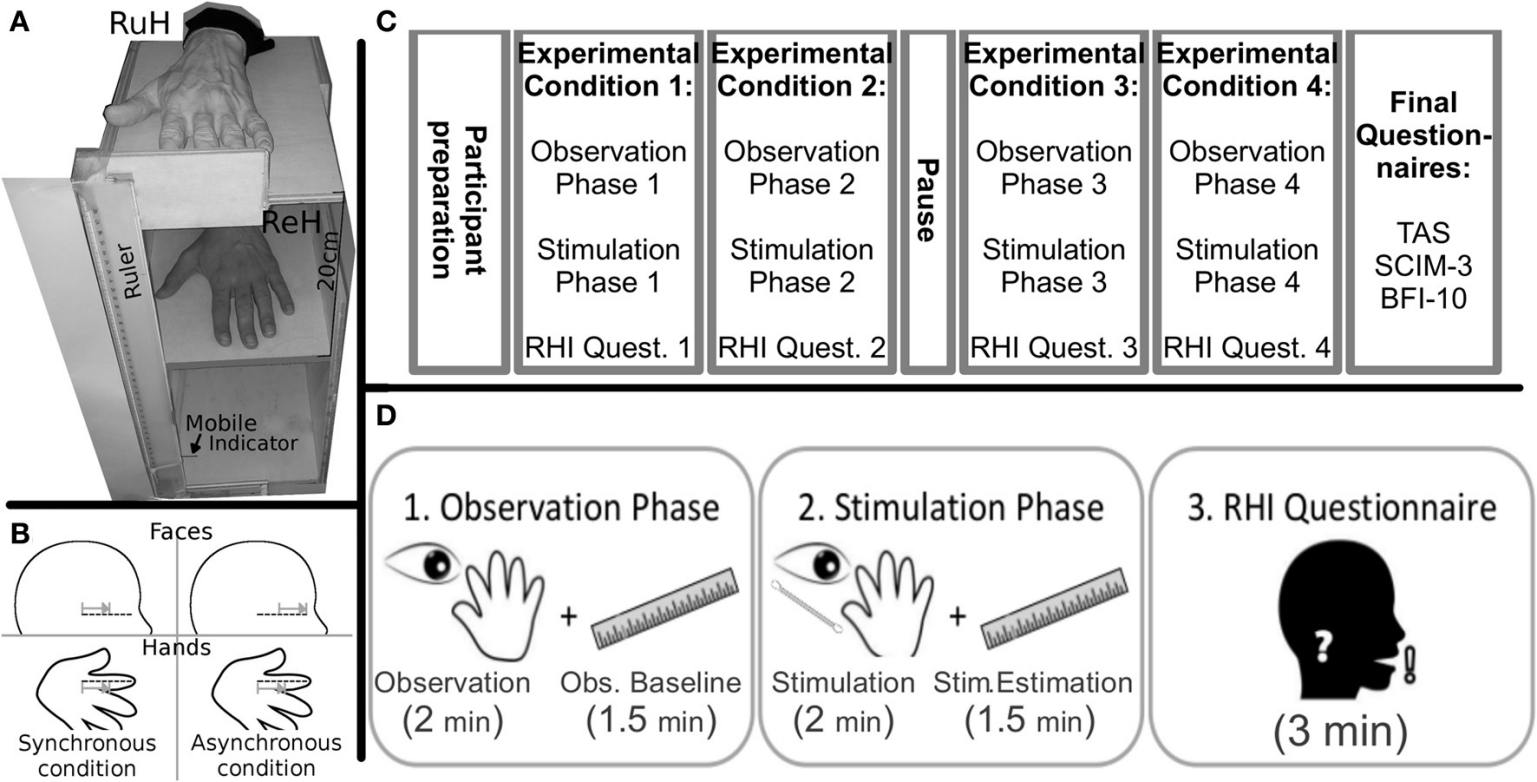
**Materials (A)** Experimental wooden box (RuH, Rubber Hand; ReH, Real Hand) the mobile indicator was always visible to the participant laterally to the box; **(B)** Graphical representation of the position and direction of the stimuli in the Synchronous and Asynchronous Face conditions. The dotted lines indicate the ideal line on the left cheek and the RH index finger. Note that in the synchronous conditions, the strokes are administered in a similar way along the ideal lines on the index finger and the cheek. In contrast, in the asynchronous condition, the relative position and/or direction of the stroke are different; (grey lines and arrows); *Experimental Timeline*. **(C)** schematic representation of the various phases of the experiment; **(D)** the sequence of the three steps (observation, stimulation and questionnaire) in each experimental condition are represented.

As shown in Figure 1A, a wooden box was built to allow the positioning of the left Real Hand and the Rubber Hand, one positioned exactly on top of the other so that they corresponded. The wooden box (H:50 cm, W:30 cm, D:40 cm) was divided into two compartments: the real hand rested upon the dividing plank inside the box, while the rubber hand was positioned on the top of the box (20 cm above the real hand). Both the participants right and left arms were hidden from view by a black cloth. The rubber hand was similar to a normal left hand. A ruler with a sliding indicator was employed so that participants could indicate the perceived position of their real hand during experimental manipulations (see below). The ruler was placed on the left side of the box with the numbers (in mm) covered so that the participants could not see them (range of shifting = 44 cm, 22 cm above and below the real hand).

#### 2.2.1. Experimental procedure

*Preparation of the participants:* Participants sat in their wheelchair (Tetraplegics and Paraplegics groups) or on a normal chair (Healthy group). The box was placed laterally to the left of each participant. The real hand was placed inside the box in a comfortable position and the rubber hand was placed more or less at the same height as the participants elbow. The participants wore special blinkers to prevent them from seeing the Q-tip approaching their cheek in the Face-conditions. The special blinkers consisted of a simple eyeglass frame with a little (5 × 4 cm) cloth attached to the left eyeglass temple. Furthermore, the box was covered with a black cloth for the whole duration of the experiment (pauses included), to avoid any additional visuo-spatial information in the experimental conditions (see below). The participants were asked to keep their hands still.

*Experimental Conditions:* The experiment was divided into four conditions (Figure 1C) involving a combination of Stimulation Type (Synchronous, Asynchronous) and Body Part Stimulated (Hand, Face). The four conditions were run in separate blocks: (i) Hand-Synchronous; (ii) Hand-Asynchronous; (iii) Face-Synchronous; (iv) Face-Asynchronous. A pause in which participants could move their real hand was allowed after two blocks. The participants left cheek and the index finger of the rubber hand were stimulated by a Q-tip in the Face-conditions and the dorsum of the participants real left index finger and the same location on the rubber hand were stimulated in the Hand-conditions. All conditions were counterbalanced across participants. Each stimulation block consisted of two phases (Figure 1D).

1. The Observation Phase.
  The participants were asked to observe the rubber hand positioned on the wooden box for 2 min. The rubber hand was then covered and participants had to estimate the vertical position of the real hand (Observation Baseline), using a verbal command to stop the mobile indicator of the ruler (moved by the experimenter) at the perceived position of their hand. When the participants stopped the mobile indicator, the experimenter recorded the position on the ruler (mm). The mobile indicator was then positioned at one of the extremes of its range as the starting position for the subsequent hand position estimation trial. The mobile indicator was thus moved in either top-down or bottom-up alternate directions. This procedure was repeated at intervals of 30 s for a total of 4 measurements (total 90 s.). The direction (top-down or bottom-up) of the first estimate was counterbalanced across participants.
2. The Stimulation Phase.
  While the participants looked at the rubber hand, a stimulation was synchronously or asynchronously administered to both the rubber hand and either the real hand or cheek of the participant. The tactile strokes were manually administered by the experimenter for 2 min by means of Q-tips. In the Hand-conditions, the rubber hand and the real hand were stimulated on dorsum of the index finger, independently of the tactile sensitivity of the real hand. In the Face-conditions, the strokes were administered to the index finger of the rubber hand and along an ideal horizontal line on the participants cheek, starting from the zygomaticus muscle (below the left eye) and moving toward the nose (see Figure 1B).

While in the Synchronous conditions the two stimulations were administered simultaneously, a temporal discrepancy was introduced in the Asynchronous conditions between the touch to the participants body part and the observed touch on the rubber hand. After stimulation, the rubber hand was covered and the participants were requested to estimate their real hand position (Stim. Estimation, Figure 1D). Subsequently, the participants answered a 6-item questionnaire (Table 2) derived from one created by Botvinick and Cohen (1998). They indicated their agreement with each item on a 10-point scale ranging from 0 (“I totally disagree”) to 10 (“I totally agree”). The first three statements were designed to capture the phenomenology of the RHI (Illusion Related Questions, IRQ), whereas the other questions were designed to be Illusion Control Questions (ICQ) (see Table 2).

**Table 2:** **The Rubber Hand Illusion questionnaire in the version for Face and Hand stimulation**.

**ID**	**Questions for the Face conditions**	**Questions for the Hand conditions**
IRQ 1	It seemed as I were feeling the touch of the Q-tip in the location where I saw the rubber hand touched	
IRQ 2	It seemed as though the touch I felt on my cheek was caused by the Q-tip touching the rubber hand	It seemed as though the touch I felt on my hand was caused by the Q-tip touching the rubber hand
IRQ 3	I felt as if the rubber hand was my hand	
ICQ 1	It felt as if my real hand was slowly drifting toward upwards	
ICQ 2	It seemed as if I might have more than one left hand or arm	
ICQ 3	It seemed as if the touch I was feeling came from somewhere between my own cheek and the rubber hand	It seemed as if the touch I was feeling came from somewhere between my own hand and the rubber hand

*The questions relating to the illusion are indicated by the letters IRQ and the control questions by ICQ*.

The whole experiment lasted about 40 min.

#### 2.2.2. Personality and absorption scales

Studies on healthy individuals suggest that the tendency to experience illusory body phenomena may be related to personality (MacLachlan et al., 2003). With the aim of exploring possible personality effects on the RHI, all participants were submitted to the Big-Five Inventory (BFI-10, Rammstedt and John, 2007) and the Tellegen Absorption Scale (TAS, Tellegen and Atkinson, 1974). The BFI-10 is a self-report interview designed to measure five dimensions of personality. The TAS (Tellegen and Atkinson, 1974) assesses the tendency to absorb others experiences, self-altering experiences and a broader trait of openness to experience.

### 2.3. Data handling

All analyses were performed using R (R Development Core Team, 2013), ggplot2 (Wickham, 2009) for graphical representations, the package lme4 ver. 1.1-5 for Mixed Linear Effects analyses (Bates et al., 2013), and α was set to 0.05. In multiple testing, significances were adjusted via false-discovery-rate procedure (FDR) (Benjamini and Hochberg, 1995).

For each experimental condition, the drift was computed by subtracting the mean of the estimations in each Observation Baseline from the Stimulation condition. In order to avoid inflation of Type I error, caused by the small sample, we used two alternative approaches that converged on similar results. First of all we dichotomised data and analysed them via log-linear models using as criterion the 95% Confidence-Interval upper-bound of all participants (drifts lower than the criterion = 0, otherwise = 1). As *post-hoc* analyses we used χ^2^ tests FDR corrected. Furthermore, a Mixed Linear Effect model was applied to drift data, without any dichotomization. The Mixed Linear Effect Model (for detailed explanations see statistics handbooks like Pinheiro and Bates, 2000; Bates, 2010), are used in previous studies (e.g., Fugard et al., 2009; Kliegl et al., 2009). For the overall analysis of the model, parametric bootstrap confidence intervals for the tests (Wald tests) and bootstrapped *p*-values were computed (see Efron and Tibshirani, 1994). FDR corrected *t*-tests were used as *post-hoc* tests.

Likert scale values in the RHI questionnaire values are organised as ordinal data. Therefore the best suited analyses are the non-parametric ones. The ICQ are questions not related to the illusion, that could be considered as a measure of the participant response bias, namely the tendency of participants to give the answers that they think could be in accord with the experimenter expectancy. This consideration is also supported by the empirical observation that in the majority of RHI experiments, if the illusion is present, the IRQ have higher values while the ICQ have lower values (Botvinick and Cohen, 1998; Aimola Davies et al., 2010; Bekrater-Bodmann et al., 2012). Therefore, to understand if an illusory effect in a condition was present, testing IRQ vs. ICQ could be an effective test. However, testing IRQ vs. ICQ or testing IRQ-ICQ against zero in a Wilxocon Signed Rank Test is mathematically equivalent (for the formula see Wilcoxon, 1945), for a demonstration please contact the first author). Therefore we adopted IRQ-ICQ as an index of the presence of the illusion. In order to understand if the illusion was present in specific conditions, we used the non-parametric Friedman's ANOVA. Because Friedman's ANOVA only allows a One-Way within-subjects analysis, initially we collapsed the Stimulation-Type and the Body-Part factors into a unique 4-level Condition, to test if there is a general effect of condition. Then we analysed the three groups (Healthy, Paraplegics and Tetraplegics) separately with the same analysis. Furthermore we tested if there are any differences among groups with a Kruskal-Wallis analysis (the non-parametric version of a One-Way between-subject ANOVA). Finally, as *post-hoc* tests, we used Wilcoxon Signed Rank tests FDR corrected.

Thus, we assessed two dependent variables for each condition, namely: (i) the proprioceptive drift, which is considered to be an index of the perceived position of the limb-in-space and (ii) the subjective report in the questionnaire, considered as an index of ownership of the rubber hand.

Personality traits (BFI-10), absorption (TAS) and functional independence (SCIM-3) were analysed to test the differences between groups and any effects on drift and IRQ-ICQ.

## 3. Results

### 3.1. Proprioceptive drift of the real hand toward the rubber hand as a subjective index of perception of the body in space

**Figure 2:**
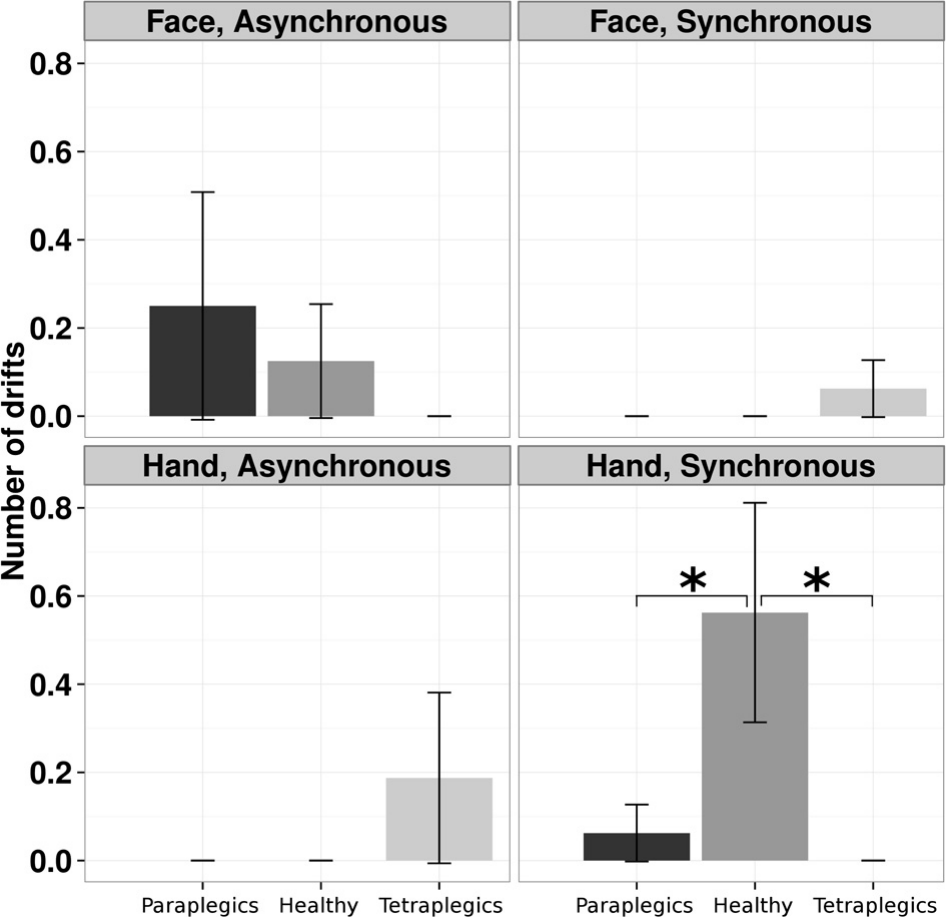
**Drifts in the position of the participants hand in the various conditions**. The mean (SE) number of drifts greater than the 95% CI upper bound for each group is reported. ^*^p < 0.05.

A log-linear model was applied to the number of drifts, with Group (Tetraplegics, Paraplegics, Healthy), Stimulation-Type (Synchronous and Asynchronous) and Body-Part (Hand, Face) as factors. The three-way interaction was statistically significant (χ^2^_(2)_ = 7.5073, *p* = 0.02). Pairwise-χ^2^-tests (FDR corrected) between groups only showed significant differences in the Hand-Synchronous condition, with more drifts in the Healthy group than in the Paraplegics group (drift number: Healthy = 9, Paraplegics = 1; χ^2^_(1)_ = 6.879, *p* = 0.05, ϕ = 14.66) and the Tetraplegics group (drift number: Tetraplegics = 0; χ^2^_(1)_ = 9.581, *p* = 0.02, ϕ = 17.30). In the Tetraplegics group, the comparison between subjects with spared hand tactile-sensitivity and subjects with sensory deficits was not significant in any of the conditions (all *p*-values > 0.56). In Figure 2 a graphical representation.

In the Mixed Linear Effect model we used as fixed factors the Stimulation-Type (Synchronous, Asynchronous), the Body Part (Hand, Face) and the Group (Healthy, Paraplegics, Tetraplegics), and Subject as random factor. The three-way interaction turned out to be statistically significant (Wald χ^2^_(2)_ = 9.43, *CI* = −6.39, 20.33, *p* < 0.01). *Post-hoc t*-tests FDR corrected were computed, comparing Synchronous vs. Asynchronous stimulation for each Body Part and Group. Only the comparison in the Healthy group in the Hand condition reached the statistical significance (*p* = 0.0125).

### 3.2. Scores in the questionnaire as a subjective index of ownership of a fake hand

For each group we performed a One-Way Friedman ANOVA on IRQ-ICQ responses, with the Stimulation-Type and Body-Part factors collapsed into a unique 4-level Condition within-subject factor. A significant effect of Condition (Friedman's χ^2^_(3)_ = 77.45, *p* < 0.0001) was found. The Friedman's tests were still significant after dividing the analysis according to the groups (Healthy group: χ^2^_(3)_ = 28.63, *p* < 0.0001; Paraplegics group: χ^2^_(3)_ = 39.14, *p* < 0.0001; Tetraplegics group: χ^2^_(3)_ = 17.46, *p* < 0.0001). A Kruskal-Wallis test was applied to the IRQ-ICQ scores for the Synchronous condition minus the IRQ-ICQ scores for the Asynchronous condition, with the Group and Body Part factors collapsed in a unique 6-levels factor. The Kruskal-Wallis test showed a statistically significant effect (Kruskal-Wallis χ^2^_(5)_ = 27.2807; *p* = 0.0001). This result indicates that the three groups have different values in the RHI questionnaire in the different conditions.

In the Tetraplegics group, Wilcoxon tests (FDR corrected) showed significantly greater values in IRQ than ICQ responses in the Hand-Synchronous (*W* = 550.5, *p* = 0.0001, *r* = 0.75), Hand-Asynchronous (*W* = 292.5, *p* = 0.026, *r* = 0.41) and Face-Synchronous conditions (*W* = 258.5, *p* = 0.024, *r* = 0.47). In the Paraplegics group, significant values were found in the Hand-Synchronous (*W* = 780, *p* < 0.0001, *r* = 1.03) and Hand-Asynchronous conditions (*W* = 425, *p* = 0.0002, *r* = 0.66). Finally, in the Healthy group the only significant effect was in the Hand-Synchronous condition (*W* = 903, *p* < 0.0001, *r* = 1.05).

**Figure 3:**
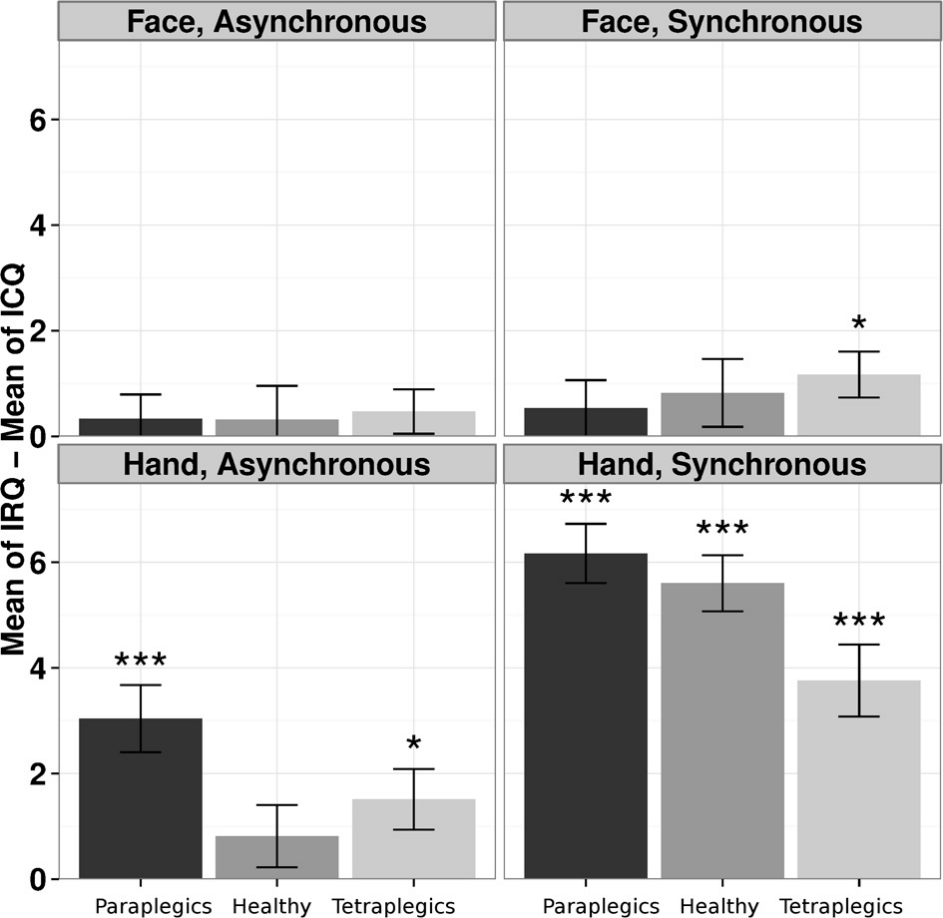
**The illusion according to participants responses to the questionnaire**. The differences between responses in IRQ and ICQ (mean values and standard errors) in the three groups are represented for each condition. ^*^p < 0.05; ^***^p < 0.001.

In the Tetraplegics group, no difference between participants with impaired or spared hand tactile sensitivity was found (all *p*-values > 0.24). For a graphical representation, see Figure 3.

### 3.3. Personality, absorption and clinical variables

TAS and BFI-10 values (see Table 3) did not differ among groups. SCIM-3 values are reported in Table 1. No statistically significant correlations were found between drifts or questionnaire responses and the TAS, the BFI-10, the SCIM-3 and the NLI.

**Table 3:** **Psychological and Personality aspects of the groups**.

**Group**	**TAS**	**Big Five Questionnaire**				
		**Extrav**.	**Agrea**.	**Consc**.	**Neur**.	**Open**.
Tetraplegics	35.38 (17.9)	7.56 (2.03)	6.81 (1.56)	7.38 (2.09)	5.13 (2.25)	7.13 (2.28)
Paraplegics	37.33 (12.49)	7.06 (2.26)	6.38 (2.13)	7.44 (2.03)	4.88 (2.68)	6.94 (1.91)
Healthy	45.06 (13.36)	6 (1.67)	6.75 (2.14)	8.31 (1.7)	5.25 (2.27)	7.69 (1.96)

*Mean (SD) values per group in the Tellegen Absorption Scale and Big Five Questionnaire. TAS, Tellegen Absorption Scale (total score); Extrav., Extraversion; Agrea., Agreeableness; Consc., Conscientiousness; Neur., Neuroticism; Open., Openness to experience*.

## 4. Discussion

This study explored whether RHI (which involves a process of integrating visual and tactile input) may constitute a reliable proxy for exploring and understanding the plasticity of bodily representations in people with SCI who suffer from massive disconnection of the body from the brain. There were three new, potentially important findings. The first is that the level of the lesion seems to influence the probability that the RHI will occur. More specifically, the more massively disconnected Tetraplegics group showed indices of ownership of the fake hand, as inferred from the questionnaire, both in the Hand (Synchronous and Asynchronous) and in the Face-Synchronous conditions. The less massively disconnected Paraplegics group showed subjective indices of RHI only in the two Hand conditions. Finally the Healthy group showed the illusion exclusively in the Hand-Synchronous condition. This picture indicates that the ownership component of RHI is related to different degrees of disconnection-related, topographic plasticity. The second finding is that the index of perception of body in space, as inferred from the drift, was found only in the Healthy group and only in the Hand-Synchronous condition. This suggests that this component of RHI is profoundly altered by somatosensory and motor body-brain disconnection. Finally, personality traits and the degree of functional autonomy in SCI do not modulate RHI.

### 4.1. Plastic influences of somatosensory de-afferentation/ motor de-afferentation on RHI

Studies on healthy people demonstrate that if they see a tactile stimulus administered to a fake hand and feel a simultaneous tactile stimulus on their own real hand (hidden from view), an illusion of incorporation of the fake hand is induced and/or the feeling that the felt tactile sensation is projected onto the rubber hand (Pavani et al., 2000; Aimola Davies et al., 2010; Haans et al., 2012). Moreover, in its canonical description, the manifestation of RHI requires not only synchronicity of stimulation but the rubber hand must also be congruent with the real one in terms of position and identity (Tsakiris and Haggard, 2005; Zopf et al., 2010). Studies also indicate that the RHI may be stronger in the vertical version (i.e., with the rubber hand positioned above the real hand) as compared to the original horizontal version (Bekrater-Bodmann et al., 2012). By adopting the vertical version of the RHI paradigm, we confirmed that in healthy subjects the phenomenal component of RHI is triggered by synchronous hand stimulation. Significantly, we demonstrated that for participants with SCI this component of RHI is greater in people with a higher level of the lesion who suffer from more massive deprivation. One likely explanation for these results has to do with evidence of functional and structural reorganization after de-afferentation of regions involved in somatosensory and motor processing (Çermik et al., 2006; Castro et al., 2007; Wrigley et al., 2009; Aguilar et al., 2010; Freund et al., 2011b; Henderson et al., 2011; Freund et al., 2013; Humanes-Valera et al., 2013; Sabre et al., 2013). Our result is in keeping with the direct demonstration of possible across-body parts remapping in people afflicted by SCI. For example, in tetraplegics who move a body part with intact representation (e.g., the tongue), the focus of neural activity in the primary motor cortex shifts toward the de-afferented upper limb representation with a strong correlation between the degree of SCI and the shift (Mikulis et al., 2002). In a similar vein, shifts of the cortical sensorimotor representations of intact body parts toward disconnected ones have been reported after SCI (Kokotilo et al., 2009). The Face-Hand illusion effect found in tetraplegics (but not in paraplegics and healthy people) may thus be interpreted as a perceptual index of topographical cortical and subcortical remapping (Freund et al., 2013). This is in keeping with what was reported in an amputee patient who underwent hands transplant, and may be the effect of co-existing hand-face representations (Farnè et al., 2002).

The increased sense of ownership of the fake hand as indicated by the questionnaire expands our previous study reporting a comparable effect in SCI and healthy subjects (Lenggenhager et al., 2012). Moreover, our study contributes to previous studies showing feeling of ownership may occur not only after synchronous stroking but also after asynchronous stroking (Rohde et al., 2011). Indeed in our study the subjective sense of ownership of the fake hand was induced in SCI groups even in the asynchronous hand stimulation condition. The fact that somatosensory deficits of the hand being stimulated did not correlate with the participants reports in the questionnaire suggests that the integrity of tactuo-proprioceptive information (likely driving bottom-up modulations) does not influence the questionnaire component of the RHI. Thus, we suggest that top-down modulations, exerted as a result of observing the fake hand, mediate the embodiment of the rubber hand and the projection of sensations onto it. This may be in keeping with studies using the mirror box illusion in which amputee patients experience ownership of a rubber hand seen in a mirror in the absence of tactile stimuli on their intact hand (Giummarra et al., 2010). In a similar vein, studies on brain damaged patients indicate that the mere sight of a rubber hand brings about a sense of incorporation of an alien hand (Fotopoulou et al., 2008; Garbarini et al., 2013).

### 4.2. Spinal cord injury abolishes changes in body perception in space induced by the RHI

RHI experiments on healthy subjects typically demonstrate robust proprioceptive drifts that have been considered a strong behavioral proxy to embodiment (Botvinick and Cohen, 1998). Interestingly however, healthy subjects may not only report the drift when they are asked to judge the position of the finger that has just been stroked, but also report the misallocation of an adjacent finger (Tsakiris and Haggard, 2005). Thus, although tactile information is very important in terms of inducing the drift, top-down modulations of bodily representations may also influence this component of RHI. In line with this, it has been suggested that the drift occurs only when the observed rubber hand is congruent in terms of posture and identity with the participants unseen hand (Tsakiris and Haggard, 2005). Measurements of the perceived localization of the participants hand before and after the various different stimulation conditions indicate that, unlike the healthy controls, the SCI subjects did not show any proprioceptive drift. This result is different from what was reported in a previous study where the perceived localization of the body in space, as indicated by the drift, was maximal in SCI patients with defective tactile sensations in the stimulated hand (Lenggenhager et al., 2012). While no ready explanation for this somewhat paradoxical result is currently available, one may hypothesize that the relative somatosensory impairment of the fingers stimulated in the Lenggenhager et al. (2012) study makes the resulting, noisy stimulation more salient. It is worth noting however, that the two studies cannot be easily compared. There is a clear difference between the two paradigms related to the position of rubber hand relative to real hand which was vertical in the present study, while horizontal in Lenggenhager et al. (2012). Moreover, the criterion used for detecting drift is here more conservative. Finally, the clinical severity of the Tetraplegics group seems to be greater in the present study. At any rate, a tentative explanation for the absence of drift found in the present study is related to the notion that, under physiological conditions, the stable representation of bodily self is dynamically updated by incoming sensory-motor information (Head and Holmes, 1911). Thus, we posit that in SCI subjects the interruption of the somatic body-brain connections may induce a bias toward a predominance of the top-down (e.g., mere sight of the rubber hand) over the bottom-up processes (e.g., tactile information from the real hand).

### 4.3. The questionnaire and the drift reveal different components of RHI

The debate about the processes underlying the RHI is still very vigorous. While the illusion was originally thought to be an effect of the dominant role of vision in intermodal integration (Botvinick and Cohen, 1998), subsequent studies suggested that it may be induced by other objects than a fake hand and thus stem from a bottom-up Bayesian perceptual learning process rather than from a process of embodiment and change in body self-representation (Armel and Ramachandran, 2003) (but see (Tsakiris and Haggard, 2005). In addition, a recent study demonstrates that the RHI can be induced by the mere observation of an object approaching the rubber hand but without touching it (Ferri et al., 2013). Studies on healthy subjects suggest that the two RHI components indicating ownership of an artificial hand and the illusory perception of the body in space (hand drift) do not go hand in hand (Rohde et al., 2011). Our data on SCI subjects provide further evidence of this dissociation between these two components of the RHI. In particular, we posit that the illusory ownership as assessed by the questionnaire may be related to mainly visual, top-down modulation while the proprioceptive drift may be based on bottom-up information processing. Thus, while post-deprivation neural plasticity may amplify illusory ownership in SCI subjects, the lack of afference and bottom-up information may cause lack of drift.

### 4.4. No effects of personality variables and functional autonomy on the strength of the RHI

Although the RHI is largely used as a direct index of body-ownership, studies demonstrate an elevated inter-individual variability in the effect (Haans et al., 2012) as well as a partial independency from bodily awareness (David et al., 2013). Moreover, the fact that the mere sight of the rubber hand triggers the RHI more than the tactile sensation does (Pavani et al., 2000; Aimola Davies et al., 2010) might suggest that personal variables, such as suggestibility, play a role in the phenomenon beyond neuroplasticity. However, no relationship between the indices of RHI and the results of the personality and susceptibility tests was found in our sample, suggesting that studies with a larger sample are necessary to demonstrate whether the absence of evidence really means that this relationship does not exist. In a previous study on SCI subjects (Pernigo et al., 2012), we demonstrated that practicing sport was useful in terms of strengthening the visual representation of upper limb body parts and contrasting the effects of somatosensory and motor deprivation. As a result we reasoned that in this study the degree of functional autonomy (which is mainly linked to the extent of the lesion) might influence the effects of the RHI. However, no correlation between these two variables was found. While this negative result may suggest that the visual perception of other people's bodies and RHI are largely independent phenomena, further study on this issue is necessary to explore the link between RHI and somatosensory and motor deprivation. Further insights about face-hand remapping may be revealed by the stroking of different body parts.

### 4.5. Qualitative reports

At the end of each experiment we asked to the participants if they felt any particular sensation that was not captured by the questionnaires or if they had any additional comments. Normally no sensations and no comments were referred, except in three cases.

One tetraplegic participant (T6) reported that in the middle of the Face Synchronous condition he started to feel the touch on the hand that was usually insensible to touch since his spinal cord lesion, 30 years earlier. At the end of the experiment he tried to touch the rubber hand with his own right hand to test whether he could feel the touch again. Unfortunately he couldnt.

Another tetraplegic participant (T5) reported that, starting from the first hand condition, every time we touched the rubber hand with the Q-tip, he felt a light pain sensation at his own hand, that he located at the rubber hand position and not at the real hand position. His real hand was insensible since the traumatic lesion of the spinal cord, 13 years earlier.

A paraplegic participant (P14), reported that, during the Face conditions, the mere vision of the rubber hand was strong enough to feel the embodiment sensation, but the tactile stimulation at the left cheek interrupted this illusion.

The subjective report of T6 seems in line with our result that in Tetraplegics also facial stimulation can evoke ownership illusion, while T5 shows the presence of illusion in both the synchronous and asynchronous Hand conditions. The participant P14, instead, suggests that, in some people, mere vision of the Rubber Hand may cause the illusion, interrupted by the tactile stimulation, similarly to what was observed by Rohde et al. (2011).

### 4.6. Limitations of the current study

Some possible limitations of this study deserve discussion. The variety of the NLI levels and in the time interval between the lesion onset and the experimental session in both the Tetraplegics and Paraplegics groups are relevant. These differences imply each individual in the same group does differ in motor, tactile and proprioceptive functions that surely have effects on neuroplastic somatosensory and motor cortical changes. For example, a person affected by paraplegia, with a T1 NLI has dramatically less control of his/her own trunk than people with a L4 lesion. These differences are even more striking in the Tetraplegics group, where a difference among C4, C5, and C6 greatly impact in the possibility of arm movements, from the complete paralysis to the possibility of motion and use of tools. It is also worth noting that more chronic SCI subjects could have learned a higher number functional strategies than a less chronic SCI subject. This should have neuroplastic consequences. Furthermore, even if there are not statistically significant differences between groups, the fact that some Tetraplegics could feel the tactile sensation of the Q-tip in the hand, while other participants could not feel it, probably have some influence on the results of this study. Finally it was not possible to have the MRI scans before and after the SCI thus our suggestions regarding the influence of lesion onset, tactile sensitivity and neuroplastic changes remain speculative.

However, finding significant effects in spite of the above reported characteristics of heterogeneity could be an indication of robustness of the effects themselves, thus our results are in keeping with the typical neuroplastic changes following SCI reported in previous literature (Bruehlmeier et al., 1998; Freund et al., 2011a,b) and seem to support the notion that motor and sensory representation of spared body parts shift toward the areas contiguous with de-efferented and de-afferented body parts.

## 5. Conclusion

This study has demonstrated that indices of ownership of a fake hand can be induced in SCI subjects and that the indication od illusory ownership over the rubber hand is more likely to occur in the presence of upper spinal levels and thus involves greater de-afferentation. In Tetraplegics the phenomenon is also induced by facial stimulation suggesting that deprivation related plasticity may occur according to somatotopic rules. Further studies are needed to understand whether plastic changes following SCI are inherently adaptive or maladaptive (Kokotilo et al., 2009; Nishimura and Isa, 2009). The absence of changes in the perceived position of the body in space confirms that these two components of the RHI may be more dissociated in SCI subjects than in healthy individuals (Rohde et al., 2011).

## Author contributions

Michele Scandola designed and performed research, analyzed data and wrote the paper. Emmanuele Tidoni contributed to design research and to write the paper. Renato Avesani and Giovanni Brunelli helped with data collection. Salvatore M. Aglioti and Valentina Moro designed research, supervised the project and contributed to write the paper.

### Conflict of interest statement

The authors declare that the research was conducted in the absence of any commercial or financial relationships that could be construed as a potential conflict of interest.
